# Bovine hypodermosis is highly prevalent in Kazakhstan: Results of a first serological study

**DOI:** 10.14202/vetworld.2023.1289-1292

**Published:** 2023-06-09

**Authors:** Christian Bauer, Marat Kuibagarov, Lyudmila A. Lider, Dinara M. Seitkamzina, Zhanbolat A. Suranshiyev

**Affiliations:** 1 Institute of Parasitology, Justus Liebig University Giessen, 35392 Giessen, Germany; 2 Department of Veterinary Medicine, S. Seifullin Kazakh Agro Technical University, 010011 Astana, Kazakhstan; 3 National Center for Biotechnology, 010000 Astana, Kazakhstan

**Keywords:** cattle, *Hypoderma*, Kazakhstan, myiasis, seroprevalence

## Abstract

**Background and Aim:**

Recent information on the occurrence of bovine hypodermosis in Kazakhstan is limited to the results of a few clinical studies in the northern and eastern regions. A first serological study aimed to obtain more data on its geographical distribution and to estimate the prevalence in this country.

**Material and Methods:**

Serum samples collected from 891 dairy cows on 30 dairy farms in eight Kazakh provinces during the winter season 2015/2016 were examined for antibodies to *Hypoderma* spp. first-stage larval antigen using a commercially available enzyme-linked immunosorbent assay (IDEXX Bovine Hypodermosis Serum Antibody Test).

**Results:**

Overall, 73.6% (95% confidence interval: 70.6%–76.5%) of the cows sampled were seropositive for *Hypoderma*, and antibody-positive cows were found in 28 of 30 farms and in seven of eight provinces.

**Conclusion:**

The results suggest a high prevalence of bovine hypodermosis in Kazakhstan, for which the socioeconomic changes in agriculture and village life following the country’s independence are considered to be responsible.

## Introduction

Hypodermosis in cattle and other bovine species (yak, buffalo, and bison) is caused by the larval stages of three *Hypoderma* species (“warble flies”): *Hypoderma bovis* (Linnaeus, 1758), *Hypoderma lineatum* (De Villers, 1789), and *Hypoderma sinense* (Pleske, 1926) [1, 2]. *Hypoderma* spp. have an annual life cycle: Adult flies deposit eggs on the hair of hosts in summer, first-stage larvae hatch from the eggs, burrow through the host’s skin, and migrate to the spinal canal (*H. bovis*) or esophagus tissue (*H. lineatum*, *H. sinense*) to overwinter. The following spring, the larvae reach the subcutaneous tissue of the back and develop into the second and third larval stages, forming characteristic subcutaneous bumps called “warbles” [1, 2]. Warbles cause suffering in cattle which, depending on the number of larvae, can result in reduced milk yield, lack of bodyweight (BWT) gain, and depreciated carcasses, as well as in considerable losses of hide quality for the leather industry. Total annual losses due to hypodermosis have been estimated at 6.5 billion rubles (approximately 220 million US dollars) in Russia [3], and losses due to damaged hides have been estimated at about 15 million US dollars per year in Northern China [4]. Hypodermosis is also a welfare issue because of the painful warbles.

Clinical studies showed that bovine hypodermosis still occurs in many regions of Central Asia, for example, in mountainous areas of South-west Siberia [5] and in Tajikistan [6]. Recent data on its occurrence in Kazakhstan are limited to results from few clinical studies in the northern and eastern regions [7, 8].

Our first serological study aimed to obtain more data on the geographical distribution and to estimate the prevalence of this parasitosis in Kazakhstan, the largest Central Asian country.

## Materials and Methods

### Ethical approval

No ethical approval was necessary for this study because the blood samples used were collected during official surveillance.

### Study period, area, and origin of samples

Serum samples used were routinely collected from all adult female cattle on each farm for the official surveillance of infectious bovine rhinotracheitis and bovine leukosis in Kazakhstan during the winter season of 2015/2016. For this study, the National Reference Center for Veterinary Medicine in Astana kindly provided 891 serum samples from 2 to 6-year-old dairy cows from 30 private households and small farms in eight oblasts (Table-1); the number of animals per holding varied between 3 and 71 depending on the herd size. In the participating villages, it is common practice in the summer for the adult cattle of all households to be turned out in the morning as a single herd onto the grassland (common property or state lands), kept there during the day and returned to their owners in the evening. Information on clinical condition of the cattle was not available; however, it was known that antiparasitic treatment was given only occasionally, if at all.

**Table-1:** Seroprevalence of *Hypoderma*-specific antibodies in dairy cows from farms in eight provinces of Kazakhstan in the winter season of 2015/2016.

Province	Number of farms positive/examined	Number of cows positive/examined	Positive cows (%)	Within-farm seroprevalence (%)
Aqtöbe	4/4	41/76	53.9	16; 62; 69; 80
Qostanai	0/1	0/25	0	0
North Kazakhstan	6/6	154/233	66.1	39; 44; 48; 76; 98; 100
Aqmola	3/3	94/113	83.2	74; 77; 95
Pavlodar	5/5	154/173	89.0	74; 77; 88; 88; 100
Qaraghandy	1/1	36/37	97.3	97
Abai^a^	2/2	95/98	96.9	93; 100
Jetisu^b^	7/8	82/136	60.3	0; 25; 28; 29; 45; 67; 95; 96
Total	28/30	656/891	73.6	--

a Part of the East Kazakhstan province until 2022,

b Part of the Almaty province until 2022.

### Serological analysis

All serum samples were stored at –20°C until use. They were examined for immunoglobulin G (IgG) antibodies to *Hypoderma* spp. first-stage larval antigen using a commercially available enzyme-linked immunosorbent assay kit (Bovine Hypodermosis Serum Antibody Test; IDEXX, Montpellier SAS, France) according to the manufacturer’s instructions. In brief, test sera, as well as positive and negative controls, were diluted 1:20, given into antigen-coated (purified extract of *H. lineatum* first-stage larvae) wells and incubated at 37°C for 1 h. After three washes, anti-bovine IgG, peroxidase conjugate was added in a dilution of 1:100, the plates were incubated at 37°C for 30 min and washed thrice. The enzyme-substrate (3,3’,5,5’-tetramethylbenzidine) solution was added, and the plates were incubated in the dark at 20°C for 20 min, the stop solution was added, and the optical density (OD) was measured at 450 nm. Finally, a corrected OD value was calculated for each test serum, considering that the positive control had a minimal OD value of 0.350 and the ratio of positive and negative OD values was at least 3.5. The result was given as a percentage; test sera with ≥ 55% were considered positive.

### Statistical analysis

Data analysis was performed using the commercially available statistical software BIAS for Windows (version 9.05; Epsilon, Hochheim, Germany). The seroprevalence, including its 95% confidence interval (CI), was calculated as the quotient of the number of seropositive animals and the total number of animals examined.

## Results

*Hypoderma*-seropositive dairy cows were found in 28 (93%) of the 30 farms surveyed and in seven of eight provinces. In total, 656 (73.6%; 95% CI: 70.6%–76.5%) of the 891 cows tested were seropositive (Table-1). The mean within-farm seroprevalence in positive farms varied from 16% to 100%, with a median of 76.9% (arithmetic mean ± standard deviation: 71.5% ± 26.9%). A comparative analysis of results in the provinces was not considered useful because only a few farms were involved.

## Discussion

Recent data on the occurrence of bovine hypodermosis in Kazakhstan are limited to results from few clinical studies in the northern and eastern regions [7, 8]. This is the first serological study of this parasitosis in the country. The study was conducted in eight provinces, home to about 49% of the country’s total dairy cow population, according to the official veterinary census [9]. The serum samples had been collected for other routine examinations, and farms were not randomly selected; therefore, the results cannot be considered statistically representative of Kazakhstan. Nevertheless, the high seroprevalence of *Hypoderma* infection in cows in 28 out of 30 farms from seven out of eight provinces suggests both a wide geographical distribution and a frequent occurrence of parasitosis in the cattle population.

The overall seroprevalence found (73.6%) is much higher than the prevalence of warbles in adult cattle previously reported from clinical studies in North or East Kazakhstan (8% [7] and 12%–23% [8], respectively). This is certainly not due to regional differences but rather to the well-known higher sensitivity of the immunological diagnostic method used: *Hypoderma*-specific antibodies can be detected in cattle approximately 6 weeks after infection with first-stage larvae of *Hypoderma* spp. and disappear a few months after the third-stage larvae have left the warbles or after successful treatment [10]. Therefore, our seropositive results indicate that the affected cattle were infected with *Hypoderma* larvae at the time of sampling or just before sampling.

The high seroprevalence can be explained by the history of livestock farming in the country. In the last decades of the 20^th^ century, bovine hypodermosis had been controlled and reduced, but not eradicated, with state support in the former Soviet Union, including its Central Asian regions [3, 11]. After the dissolution of the Soviet Union and Kazakhstan’s independence in 1991, the agricultural system was restructured, leading to major economic and social problems: Collective farms (“kolkhozes”) were privatized and large-scale state subsidies were stopped [12]. This also affected the systematic control of hypodermosis, which was discontinued [13]. There have also been major demographic changes, such as increased rural-urban migration, especially among young people, and ethnic emigration and immigration (emigration of Russians and other minorities from Kazakh territory, immigration of people of Kazakh origin from abroad) [14]. After independence, veterinary medicine was no longer a priority in the country’s professional sphere. There are only a few well-qualified veterinarians, some of whom are employed in livestock farming [12]. The number of cattle in Kazakhstan fell rapidly from 9,600,000 heads in 1991 to 4,100,000 heads in 2000. Since then, there has been a gradual recovery, and in 2021 the national cattle population comprised approximately 8,200,000 heads, including 4,240,000 dairy cows, according to the official veterinary census [9]. Today, the majority of dairy cows in the country are kept by private households for subsistence purposes (51.4%) and on small farms (41.5%), while only 7.1% are kept on large agricultural enterprises [9]. However, small-scale livestock holders are generally poor in Kazakhstan [15], and most have little or no knowledge of useful preventive measures. Systematic targeted treatments are mostly applied on large enterprises but only sporadically, if at all, on small farms. These circumstances are of course responsible for the continued presence of *Hypoderma* spp. in the country.

However, bovine hypodermosis can be successfully controlled and eradicated from an area. If the control is mandatory or coordinated at a regional level, and appropriate compounds are used strictly for all cattle on all farms, eradication would be possible within a few years [16]. Therefore, province-wide campaigns aimed at eradicating this economically important parasitosis should be launched to improve the health and performance of cattle and to increase the economic income of farmers. Macrocyclic lactones, such as ivermectin or eprinomectin, are currently the first-choice compounds because of their near 100% efficacy against the *Hypoderma* first-stage larvae [1, 17]. A proven method of treatment is the application of a “microdose” of ivermectin (2 μg/kg BWT subcutaneously or 2.5 mg/kg BWT pour-on) given to all cattle in a region in late autumn [16]. Given the current socioeconomic conditions in Kazakhstan and other Central Asian countries, this cost-saving method could be of particular interest to poor smallholders.

## Conclusion

A limitation of the study is the examination of cattle from a small number of farms. However, our serological results from several regions in Kazakhstan suggest a high prevalence of bovine hypodermosis, which may be a consequence of the socioeconomic changes in agriculture and village life following the country’s independence. Based on this assumption, the relevant authorities should initiate province-wide campaigns to eradicate this parasitosis by means of appropriate control measures. In addition, cattle from all farms in an affected region should be tested after the grazing period to obtain an accurate assessment of both the prevalence of the parasitosis and the effectiveness of the control measures implemented.

## Authors’ Contributions

CB: Planned the study and drafted and revised the manuscript. MK, LAL, DMS, and ZAS: Performed the serological examinations and analyzed the data. All authors have read, reviewed, and approved the final manuscript.

## References

[1] Colwell D.D, Hall M.J.R, Scholl P.J, Colwell D.D, Hall M.J.R, Scholl P.J (2006). A synopsis of the biology, hosts, distribution, disease significance and management of the genera. The Oestrid Flies:Biology, Host-Parasite Relationships, Impact and Management.

[2] Otranto D, Paradies P, Testini G, Lia R.P, Giangaspero A, Traversa D, Colwell D.D (2006). First description of the endogenous life cycle of *Hypoderma sinense* affecting yaks and cattle in China. Med. Vet. Entomol.

[3] Glazunova A.A, Kustikova O.V, Lunina D.A, Ilyasov P.V (2019). Bovine hypodermatosis:Diagnosis, treatment and prevention (review). Russ. J. Parasitol.

[4] Yin H, Ma M, Yuan G, Huang S, Liu Z, Luo J, Guan G (2003). Hypodermosis in China. J. Anim. Vet. Adv.

[5] Saitov V.R (2005). Arachno-Entomological Fauna of Cattle in the Mountainous Areas of South Western Siberia. Candidate Medicine Veterinary.

[6] Sodatkhonova D.A, Khudoidodov B.I, Razikov Sh.Sh (2021). Cattle hypodermatosis in south Tajikistan. Theory Pract. Parasit. Dis. Control.

[7] Okunev A.M (2019). The specific features of subcutaneous warble fly development in cattle in Northern Kazakhstan. Dairy Newsl.

[8] Ikimbayeva N.A, Duyssembaev S.T, Shabdarbayeva G.S (2020). Comparative indicators of the distribution of hypodermatosis in different areas of eastern Kazakhstan region in recent years. Scientific and Practical Journal of Zhangir Khan West Kazakhstan Agrarian-Technical University.

[9] Anonymous (2023). Statistics of Agriculture, Forestry, Hunting and Fisheries. Key Indicators of Livestock Development in the Republic of Kazakhstan.

[10] Boulard C, Colwell D.D, Hall M.J.R, Scholl P.J (2006). Hypodermatinae host-parasite interactions. The Oestrid Flies:Biology, Host-Parasite Relationships, Impact and Management.

[11] Fayenova Y.R (2019). Epizootological monitoring of bovine hypodermatosis in 2015–2019. Russ. J. Parasitol.

[12] Tazhibaeva S, Musabekov K, Yesbolova A, Ibraimova S, Mergenbayeva A, Sabdenova Z, Seidahmetov M (2014). Issues in the development of the livestock sector in Kazakhstan. Procedia Soc. Behav. Sci.

[13] Baizhanov M, Berkinbay O (2004). Hypodermosis of domestic and wild animals of Kazakhstan. Chin. J. Vet. Parasitol.

[14] Dufhues T, Buchenrieder G, Sun Z (2021). Exploring policy options in regulating rural-urban migration with a Bayesian network:A case study in Kazakhstan. Eur. J. Dev. Res.

[15] Hauck M, Artykbaeva G.T, Zozulya T.N, Dulamsuren C (2016). Pastoral livestock husbandry and rural livelihoods in the forest-steppe of east Kazakhstan. J. Arid Environ.

[16] Boulard C, Alvinerie M, Argente G, Languille J, Paget L, Petit E (2008). A successful, sustainable and low-cost control-programme for bovine hypodermosis in France. Vet. Parasitol.

[17] Lia R.P, Rehbein S, Giannelli A, Fankhauser B, Otranto D (2019). Longrange®(eprinomectin 5% w/v extended-release injection) efficacy against *Hypoderma lineatum* in an endemic area in Southern Italy. Parasit. Vectors.

